# Assessment of adverse events among healthcare workers following the Janssen COVID-19 vaccine in Tigray, Ethiopia

**DOI:** 10.1038/s41598-024-53561-8

**Published:** 2024-02-08

**Authors:** Bisrat Tesfay Abera, Hale Teka, Ephrem Berhe, Marta Abrha Gebru, Dawit Zenebe, Hiluf Ebuy Abraha, Abraha Hailu

**Affiliations:** 1 Department of Internal Medicine, School of Medicine, Mekelle University, Mekelle, Tigray Ethiopia; 2 Department of Obstetrics and Gynecology, School of Medicine, Mekelle University, Mekelle, Tigray Ethiopia; 3 Department of Epidemiology, School of Public Health, Mekelle University, Mekelle, Tigray Ethiopia; 4 Department of Biostatistics, School of Public Health, Mekelle University, Mekelle, Tigray Ethiopia; 5 Arnold School of Public Health, University of South Carolina, Columbia, USA

**Keywords:** Immunology, Health care, Medical research, Risk factors

## Abstract

Apart from the inequality in vaccination, war zones and areas where communication is disrupted are affected by myths and misconceptions about COVID-19 vaccines, heightening vaccine hesitancy. Local data on adverse events of the vaccines and their mildness can increase confidence and acceptance of the vaccines in the respective population. In areas of conflict and communication blackouts, the perception of the vaccines by health workers is of paramount importance as public health recommendations may not reach the public. Therefore, the scientific evaluation of adverse events following COVID-19 vaccination in such areas is invaluable. This cross-sectional, facility-based study was conducted using a structured, interviewer-administered questionnaire to assess the adverse events experienced by healthcare workers who received the Janssen COVID-19 vaccine. The sample was divided proportionally to the number of vaccinated healthcare workers for the different healthcare professions, and participants were then randomly selected from each profession. Prior to data collection, a pilot test was conducted with 5% of the sample size outside the selected hospital. The study was conducted using a structured questionnaire completed by an interviewer to assess adverse events in 442 healthcare workers who had received the Janssen COVID-19 vaccine between July 11 and 25, 2022. The study period was from August 15 to September 15, 2022. A significant number of healthcare workers [366 (83.3%); 95% CI 79.5%, 86.5%] experienced at least one adverse event. Nearly 90% of participants reported that the adverse events were mild to moderate. Pain at the injection site [307 (69.5%); 95% CI 65.0%, 73.6%] and headache [247 (55.9%); 95% CI 51.2%, 60.4%] were the most common local and systemic adverse events, respectively. Two HCWs experienced anaphylactic reaction. Younger age was significantly associated with the occurrence of adverse events. We deciphered that the adverse events reported by the study participants were not different from the typically occurring vaccine-related adverse reactions, and therefore concluded that post-vaccination reactions in healthcare workers were minor. Although vaccination in Tigray is currently stalled due to the siege, responsible stakeholders should develop a mechanism to track population-wide adverse events once the vaccines start to rollout.

## Introduction

Mass vaccination is the most promising means of combating respiratory diseases caused by the severe acute respiratory syndrome coronavirus-2 (SARS-CoV-2). To date, the US Food and Drug Administration (FDA) has granted emergency use authorization for the Pfizer-BioNTech (BNTb262), Moderna (mRNA-1273), and Janssen COVID-19 (Ad26.COV2.S) vaccines^1^. Phase 3 clinical trials have shown that Pfizer-BioNTech and Moderna are 95% and 94.1% effective, respectively, in preventing coronavirus disease 2019 (COVID-19)^2,3^. Janssen’s COVID-19 vaccine and other adenovirus-based vaccines have also achieved good results in preventing the infection. The efficacy of the Janssen COVID-19 vaccine in preventing severe critical illness is 77% at least 14 days after administration, and increases up to 85% at least 28 days after administration^4^. In general, clinical studies have shown that COVID-19 vaccines have an efficacy of 70.4–95% in preventing symptomatic COVID-19 infection^5^. As of January 2023, more than 13 billion COVID-19 vaccinations have been administered worldwide. However, the distribution is lopsided, with less privileged countries such as those in Africa lagging behind in vaccination coverage^6^.

Despite the success of vaccines, concerns about adverse events (AEs) have been a major barrier to vaccination worldwide^7^. According to the definition of Herve et al., vaccine reactogenicity is “a subset of reactions that occur soon after vaccination and are a physical manifestation of the inflammatory response to vaccination^8^, while the WHO defines AEs as “any untoward medical occurrence which follows immunization and which does not necessarily have a causal relationship with the usage of the vaccine”^9^.

In addition to unavoidable mild and transient AEs such as local pain/redness/swelling at the injection site, and general side effects such as fatigue, muscle/joint pain, headache, chills/fever and nausea^10^, several myths and misconceptions related to vaccines, such as associated disabilities like infertility, COVID-19 infection, DNA mutation and death, pose a serious challenge^11^. A strong antipathy towards vaccines was observed following a worldwide warning due to cases of serious AEs such as thrombosis with thrombocytopenia syndrome (TTS) associated with Janssen's COVID-19 vaccine and other COVID-19 vaccines^12^. This forced the FDA to briefly suspend Janssen's COVID-19 vaccine^13^. However, this stance was immediately reversed as the vaccine performed well in preventing severe disease, and TTS and associated deaths were rare, affecting only eight people per 8 million doses^14^.

Areas where communication is interrupted are most affected by hearsay about vaccinations. The difficulty of accessing scientifically sound information and the disintegration of the health information system make it difficult to correct erroneous conclusions about vaccines^15^. The resulting reluctance to vaccinate is leading to the unchecked spread of COVID-19 infections^16^. The impact is even worse in war zones which already suffer from vaccine inequity^17^.

Tigray, a region in northern Ethiopia, has been largely excluded from COVID-19 vaccination campaigns due to a war that broke out in November 2020 and a subsequent siege imposed in June 2022^18^. Unpublished data from the region’s regional health office shows that only 2% of the population had received a single dose of the AstraZeneca vaccine in around May 2021. After that, only the residents of Mekelle, the capital of Tigray, were vaccinated in a two-week campaign from July 11 to July 25, 2022. The communication blackout and the widespread destruction of the health information system in the region are the biggest obstacles in debunking the myths and misconceptions surrounding the COVID-19 vaccines that are preventing the trickle of vaccines that have arrived in Tigray from reaching the population^19^.

Healthcare workers (HCWs) play an indispensable role in building public confidence in vaccines. Patients also rely on HCWs for information about vaccine-related AEs^20^. Therefore, vaccine hesitancy is particularly high when HCWs, who are expected to lead the campaign, are reluctant^21^. This not only puts a strain on vaccination campaigns, but also leads to significant morbidity and mortality as unvaccinated professionals transmit the virus to the most vulnerable during patient care^16^. Although the Janssen COVID-19 vaccine is currently being withdrawn by the Food and Drug Administration (FDA) at the request of the manufacturer due to expiration of the license and lack of demand in May 2023, it is considered necessary to provide local data on AEs of the vaccine^22^. Conducting a Janssen COVID-19 vaccine AEs evaluation in HCWs is critical for several reasons. It is important to understand the AEs of the vaccine in different global and regional contexts, taking into account factors such as population demographics and healthcare infrastructure. Conducting global, regional and local studies helps to identify specific patterns of AEs, differences in reporting, and the influence of HCWs characteristics on vaccine reactions. By synthesizing the information from the study, we can make informed decisions about vaccination strategies, risk communication, and targeted interventions related to AEs among HCWs at the local level (Supplementary Information).

Therefore, it is essential to assess the prevalence of AEs in HCWs after vaccination to reduce any barriers to vaccination. However, reports of AEs are mostly concentrated in industrialized countries. There are hardly any reports from Africa and war zones^23^.

The war torn Tigray also lacks data on AEs related to COVID-19 vaccines including the Janssen COVID-19 vaccine. Combined with the communication breakdown and the collapse of the health information system, this leads to low vaccination coverage, hence, a study on this issue is needed. We hypothesized that a substantial proportion of HCWs will experience AEs, most of which will be mild in nature, such that avoiding the vaccine would not be justified. Therefore, the aim of this study was to investigate the magnitude and severity of AEs following vaccination with the Janssen COVID-19 vaccine among HCWs at Ayder Comprehensive Specialized Hospital (ACSH), Tigray, Ethiopia.

## Materials and methods

### Study design

This cross-sectional, facility-based study was conducted using a structured, interviewer-administered questionnaire to assess the AEs experienced by HCWs who received the Janssen COVID-19 vaccine. The questionnaire was designed to collect data on sociodemographic characteristics, comorbidity, status of previous COVID-19 infection, previous vaccination against COVID-19, AEs related to the Janssen COVID-19 vaccine, timing of AEs, self-assessed severity of AEs, duration of AEs, and treatment required for the AEs. The data was collected by six trained physicians.

A pretest was conducted outside the selected hospital prior to data collection. There was strict monitoring during data collection.

### Study setting and participants

The study was conducted at ACSH from August 15, 2022 to September 15, 2022 in HCWs who had received the Janssen COVID-19 vaccine from July 11 to July 25, 2022. ACSH is a tertiary care hospital in Mekelle, Tigray, Northern Ethiopia. ACSH provides a comprehensive range of specialized and general healthcare services to more than 9 million people in a catchment area that includes Tigray, Eritrea, Afar, and northwestern parts of the Amhara region. It also serves as a teaching and research hospital for the University of Mekelle. It currently employs around 2088 HCWs (according to the University Hospital Human Resources Department as of July 2022).

Based on a prevalence of 50% of AEs to COVID-19 vaccines, a confidence level of 95%, a type I error of 5%, and a non-response rate of 15%, the sample size was set at 442.

**Figure 1:**
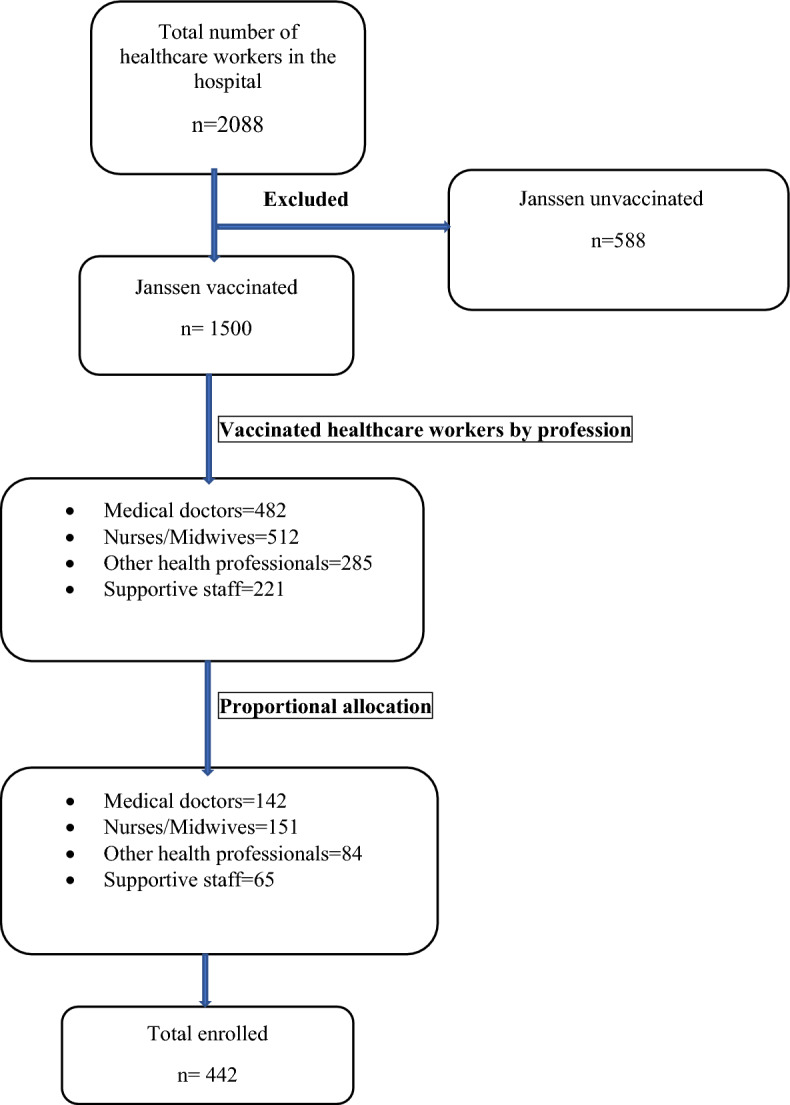
Sampling procedures used among HCWs, Tigray, Northern Ethiopia, 2022.

Information was obtained from the hospital registry to identify HCWs who had received the Janssen COVID-19 vaccine. There were 1500 vaccinated HCWs. Broken down by profession, there were 482, 512, 285, and 221 doctors, nurses/midwives, other healthcare professionals, and ancillary staff, respectively. The sample was divided proportionally according to the number of vaccinated HCWs in each profession. Participants were then randomly selected until the allocated sample for each occupational group was reached (Fig. 1).

### Measurements

The outcome of this study was the identification of AEs following vaccination with the Janssen COVID-19 vaccine and factors associated with the occurrence of AEs. Sociodemographic characteristics, comorbidity, body mass index, status of previous COVID-19 infection, history of previous vaccination against COVID-19, history of allergic reaction to medication, current COVID-19-like symptoms, history of vaccination with the AstraZeneca vaccine, and history of vaccination with other non-COVID-19 vaccines within the last 6 months were independent variables.

- Adverse event: any medical event following vaccination that is not necessarily causally related to the use of the vaccine^24^;
- Local AEs: Symptoms that occur at or near the injection site of the vaccine;
- Systemic AEs: Symptoms occurring at a site distant from the injection site;
- Healthcare workers: all paid and unpaid individuals who work in a healthcare setting (e.g. doctors, nurses, midwives, laboratory staff, pharmacy staff, anesthetists , radiology staff, health officers , facility and maintenance staff, cleaners, security staff, clinical trainees, volunteers, etc.).
- Severity of AEs: The severity of symptoms was scaled according to the medical staff's self-reported perception of the severity of AEs.

### Data analysis

The data was entered into EpiData and then analyzed using the statistical software Stata version 16. After cleaning the data, we performed descriptive statistics with frequency and percentage. The mean with standard deviation (SD) was estimated for non-skewed continuous variables, while the median with interquartile range (IQR) was used for skewed continuous variables. For categorical variables, comparisons were made using the Chi-square test for association. The *t*-test for independent samples was used to compare continuous variables between different groups.

Based on a p-value of less than 0.25 in the bivariate analysis and/or the clinical significance of some independent variables from previous work in the literature, the variables were included in the multivariable logistic regression to determine whether there was a significant association between each independent variable and the occurrence of AEs after vaccination. To control for potential confounding factors, the analysis was adjusted for the following variables: Age, gender, comorbidity, previous COVID-19 test result, history of allergic reaction, previous vaccination history, and COVID-19-like symptoms. Independent variables that were associated with AEs and had a p-value of less than 0.05 were considered statistically significant. Multicollinearity diagnostics were performed, and all variables included in the final model were noncollinear with each other. We performed the Hosmer–Lemeshow goodness-of-fit test, which showed that our model was a good model for the fitted variables.

### Ethical consideration

Ethical approval was granted by the institutional review board of Mekelle University, College of Health Sciences (IRB). Participants were informed of the purpose of the study. The anonymity of the study participants was guaranteed, and the authors had no access to individual information.

### Institutional review board statement

The study was conducted in accordance with the amended Declaration of Helsinki that it was approved by the Institutional Review Board of Mekelle University (IRB) with IRB number of 1992/2022.

### Informed consent statement

Written informed consent was obtained from all study participants.

## Results

### Sociodemographic and clinical characteristics

A total of 442 HCWs were included in the study with a response rate of 100%. The average age of the study participants was 31.1 [SD = 6.9]. Males accounted for 259 (58.6%) of the recruits. Only 33 (7.5%) had at least one comorbidity. Asthma 12 (2.7%), hypertension, eight (1.8%), and human immunodeficiency virus five (1.3%) were the most common comorbidities. Of the study participants, 24 (5.4%) reported having an allergy to various medications.

Approximately 266 (60.2%) of the study participants had received a dose of the AstraZeneca vaccine that had been administered to the hospital staff one year prior to the current study period. A total of 77 (17.4%) HCWs reported a history of laboratory-confirmed COVID-19 infection (Table 1).

**Table 1:** Demographic and clinical characteristics of the study participants, Ayder comprehensive specialized hospital, Mekelle, Tigray, Northern Ethiopia, 2022 (n = 442).

Characteristic	Total (n = 442)	Reported side effect		P-value
		Yes (n = 368)	No (n = 74)	
Age in years [mean (SD)]	31.1 (6.9)	31.8 (6.8)	33.7 (7.3)	0.03
Sex, n (%)				
Male	259 (58.6)	218 (59.2)	41 (55.4)	0.541
Female	183 (41.4)	150 (40.8)	33 (44.6)	
Occupation, n (%)				
Medical doctor	142 (32.1)	118 (32.0)	24 (32.4)	0.310
Nurse	151 (34.2)	120 (32.6)	31 (41.9)	
Other health profession^a^	84 (19.0)	72 (19.6)	12 (16.2)	
Support staff^b^	65 (14.7)	58 (15.8)	7 (9.50)	
Religion, n (%)				
Christian	424 (95.9)	352 (95.6)	72 (97.3)	0.542
Muslim	12 (2.7)	10 (2.8)	2 (2.7)	
Other	6 (1.4)	6 (1.6)	0 (0.00)	
Marital status, n (%)				
Single	199 (45.0)	173 (47.0)	26 (35.1)	0.216
Married	228 (51.6)	182 (49.5)	46 (61.2)	
Divorced	12 (2.7)	10 (2.7)	2 (2.7)	
Widowed	3 (0.70)	3 (0.80)	0 (0.00)	
Educational status, n (%)				
Higher education	402 (91.0)	334 (90.8)	68 (91.9)	0.757
Secondary and below	40 (9.0)	34 (9.2)	6 (8.1)	
Body mass index, n (%)				
Underweight	99 (22.4)	84 (22.8)	15 (20.3)	0.278
Healthy weight	309 (69.9)	259 (70.4)	50 (67.6)	
Over weight	34 (7.7)	25 (6.8)	9 (12.1)	
History of allergy, n (%)				
Yes	24 (5.4)	21 (5.7%)	3 (4.0)	0.567
No	418 (94.6)	347 (94.3)	71 (96.0)	
Current medication intake, n (%)				
Yes	29 (6.6)	23 (6.2)	6 (8.1)	0.556
No	413 (93.4)	345 (93.8)	68 (91.9)	
Comorbidity, n (%)				
Yes	33 (7.5)	26 (7.10)	7 (9.5)	0.475
No	409 (92.5)	342 (92.9)	67 (9.05)	
Previous positive COVID-19 test, n (%)				
Yes	77 (17.4)	60 (16.3)	17 (23.0)	0.168
No	365 (82.6)	308 (83.7)	57 (77.0)	
Previously vaccinated for AstraZeneca, n (%)				
Yes	266 (60.2)	222 (60.3)	44 (59.5)	0.889
No	176 (39.8)	146 (39.7)	30 (40.5)	

*SD* standard deviation. ^a^Doctor of dental surgery, pharmacy, laboratory, radiology, physiotherapy, anesthesiology, clinical psychiatry, clinical pathology, biomedicine, health officer, clinical dentistry, environmental health. ^b^Cleaner, security, health education specialist, HMIS, occupational health, ART adherence worker, porter, oxygen attendant, social worker, security guard, ambulance driver, hospital data encoders.

### Adverse events following Janssen COVID-19 vaccine

Of the 442 HCWs who participated in this study, 368 (83.3%) reported having at least one AE [95% CI 79.5%, 86.5%]. Local AEs occurred in 310 (70.1%) [95% CI 65.7%, 74.2%], while systemic AEs were observed in 309 (69.9%) [95% CI 65.5%, 74.0%].

The most common local and systemic AEs were pain at the injection site and headache, respectively. Although four (0.9%) [95% CI 0.3%, 2.3%] of the HCWs reported swelling of the eyes and face, only two (0.45%) [95% CI 0.1%, 1.0%] of the study participants reported an anaphylactic reaction (one male and one female). Transient arm weakness at the injection site was reported by one (0.23%) [95% CI 0.03%, 1.6%] HCW (Table 2).

**Table 2:** Adverse events following vaccination with Janssen COVID-19 vaccine among the participants, Ayder comprehensive specialized hospital, Mekelle, Tigray, Northern Ethiopia, 2022 (n = 442).

AEs	Frequency (%)	95% CI
At least one AE	368 (83.3)	79.5, 86.5
Local adverse event	310 (70.1)	65.7, 74.2
Pain	307 (69.5)	65.0, 73.6
Erythema	45 (10.2)	7.7, 13.4
Pruritus	21 (4.7)	3.1, 7.2
Swelling	63 (14.2)	11.3, 17.8
Rash	9 (2.0)	1.1, 3.9
Discharge	3 (0.70)	0.2, 2.1
Enlarged lymph node	15 (3.40)	2.0, 5.5
Systemic adverse event	309 (69.9)	65.5, 74.0
Headache	247 (55.9)	51.2, 60.4
Chills	193 (43.7)	39.1, 48.3
Fever	191 (43.2)	38.6, 47.9
Fatigue	231 (52.3)	47.6, 59.9
Arthralgia	175 (39.6)	35.1, 44.2
Myalgia	169 (38.2)	33.8, 42.8
Dizziness	116 (26.2)	22.3, 30.5
Nausea	37 (8.4)	6.1, 11.3
Vomiting	10 (2.3)	1.2, 4.1
Diarrhea	9 (2.0)	1.1, 3.9
Shortness of breath	5 (1.1)	0.5, 2.7
Chest pain	14 (3.2)	1.9, 5.3
Transient arm weakness	1 (0.23)	0.03, 1.6
Eye swelling	4 (0.90)	0.3, 2.3
Facial swelling	4 (0.90)	0.3, 2.3
Pharyngeal swelling	3 (0.70)	0.2, 2.1
Lip swelling	1 (0.23)	0.03, 1.6
Sleep disturbance	1 (0.23)	0.03, 1.6
Body itching	1 (0.23)	0.03, 1.6
Body rash	1 (0.23)	0.03, 1.6
Anaphylactic reaction	2 (0.45)	0.1, 1.0

*AEs* adverse events.

### Severity, management, and timing of adverse events

**Figure 2:**
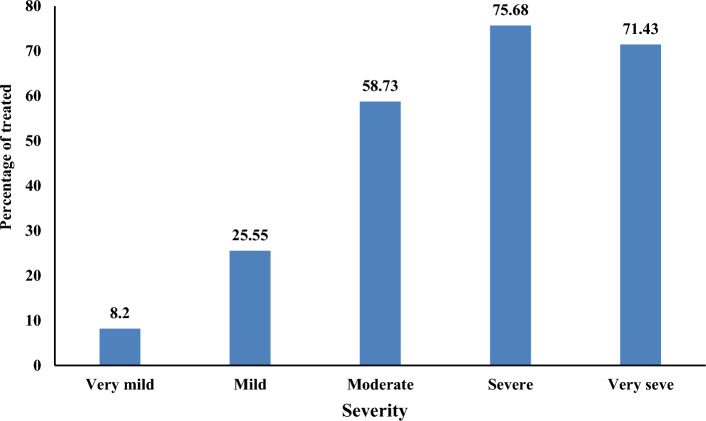
Relationship between degree of severity of symptoms and the need for treatment, Tigray, Northern Ethiopia, 2022 (n = 442).

When asked about the severity of the symptoms, 137 (37.2%) [95% CI 32.4%, 42.3%] participants stated that their symptoms were mild. AEs required treatment in 147 (40%) [95% CI 35%, 45%] of the participants. Of those who received treatment, 95 (64.6%) [95% CI 56.5%, 72.0%] were taking over-the-counter medications. The more severe the symptoms, the more often participants sought treatment: 75.68% and 71.43 of the participants with severe and very severe symptoms, respectively, sought treatment (Fig. 2).

Of the 132 participants who had previously developed AEs after vaccination with the AstraZeneca vaccine, 67 (50.8%) [95% CI 42.2%, 59.2%] perceived AEs as more severe after vaccination with Janssen COVID-19 than the AstraZeneca vaccine.

**Figure 3:**
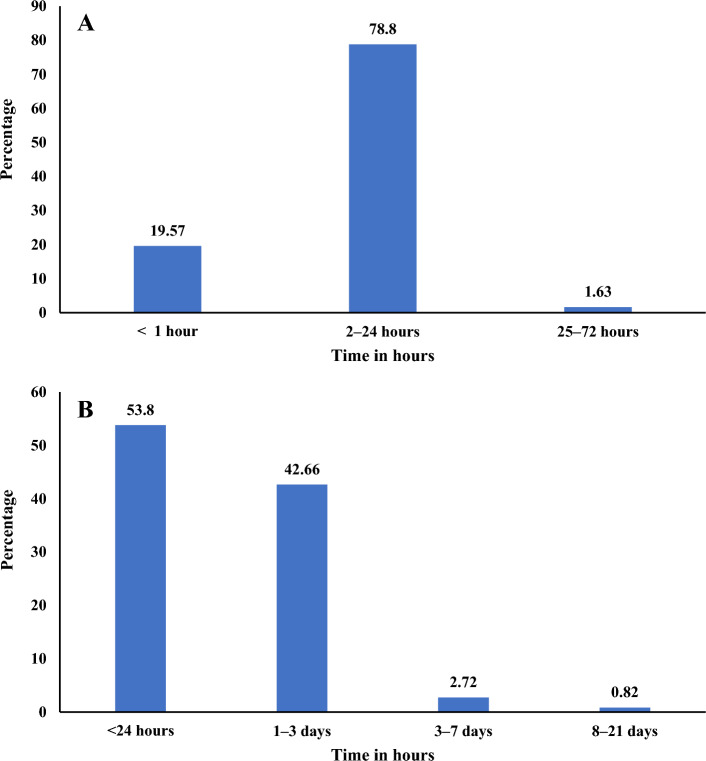
Time of onset and duration of AEs, Tigray, Northern Ethiopia, 2022 (n = 442). (**A**) Time to onset of AEs (h). (**B**) Duration of AEs (h).

Most participants (78.8%) experienced side effects in the period of 2–24 h. The symptoms disappeared in 53.8% of the participants within 24 h. Only in 0.82% of the participants did the symptoms last longer than 7 days (Table 3; Fig. 3).

**Table 3:** Severity, management and timing of adverse events following vaccination with Janssen COVID-19 vaccine among the participants, Ayder comprehensive specialized hospital, Mekelle, Tigray, Northern Ethiopia, 2022 (n = 442).

Characteristic	Frequency (%)	95% CI
Previously vaccinated with AstraZeneca	266 (60.2)	55.5, 64.6
Had adverse events following AstraZeneca	132 (49.6)	43.6, 55.6
Perceived severity of adverse event (Janssen vs AstraZeneca)		
More severe	67 (50.8)	42.2, 59.2
Similar	37 (28.0)	21.0, 36.4
Less severe	28 (21.2)	15.0, 29.1
Perceived adverse event severity		
Very mild	61 (16.6)	13.1, 20.7
Mild	137 (37.2)	32.4, 42.3
Moderate	126 (34.2)	29.5, 39.2
Severe	37 (10.1)	7.3, 13.6
Very severe*	7 (1.9)	0.9, 3.9
Side effect required treatment	147 (40.0)	35.0, 45.0
Type of treatment required		
Home based treatment	44 (30.0)	23.0, 37.9
Over the counter medication	95 (64.6)	56.5, 72.0
Consultation with a healthcare professional	5 (3.4)	1.4, 8.0
Visit to the emergency room^a^	3(2.0)	0.6, 6.2
Onset of symptoms after vaccination [median (IQR)], in hours	6 (9)	7.6, 9.3
Duration of symptoms [median (IQR)], in hours	24 (30)	32.6, 40.8

*Four had urticaria, two developed anaphylactic reaction while one reported transient arm weakness.

^a^Two with anaphylactic reaction and one with urticaria.

*IQR* interquartile range.

### Factors associated with adverse events following Janssen COVID-19 vaccination

Eight variables (age, gender, comorbidity, previous positive COVID-19 test, allergy history, previous AstraZeneca vaccination, previous uptake status of other vaccines, and COVID-19-like symptoms before vaccination) were included in the final model.

After adjusting for potential confounders, only age was found to be a statistically significant predictor of AEs: With an increase in age by one year, the probability of suffering AEs decreased by 4.0% (AOR = 0.96, 95% CI 0.92%, 0.98%) (Table 4).

**Table 4:** Factors associated with adverse event following vaccination with Janssen COVID-19 vaccine, Ayder comprehensive specialized hospital, Mekelle, Tigray, Northern Ethiopia, 2022 (n = 442). Significant values are in bold.

Variables	COR (95% CI)	AOR (95% CI)	P-value
Age in years	0.96 (0.93, 0.98)	0.96 (0.92, 0.98)	**0.038**
Sex			
Male	1.17 (0.71, 1.93)	1.15 (0.68, 1.93)	0.594
Female	1	1	
Comorbidity			
Yes	0.73 (0.30, 1.74)	1.00 (0.38, 2.61)	0.997
No	1	1	
Previous positive COVID-19 test			
Yes	0.65 (0.35, 1.20)	0.63 (0.33, 1.17)	0.142
No	1	1	
History of allergic reaction			
Yes	1.43 (0.41, 4.93)	1.58 (0.45, 5.58)	0.479
No	1	1	
Previously received AstraZeneca vaccine			
Yes	1.04 (0.62, 1.72)	1.12 (0.66, 1.91)	0.653
No	1	1	
Received other vaccines within 6 months			
Yes	1.00 (0.22, 4.69)	1.23 (0.26, 5.86)	0.793
No	1	1	
COVID-19 like symptoms just before vaccination			
Yes	1.36 (0.62, 6.24)	1.42 (0.63, 3.19)	0.393
No	1	1	

## Discussion

By conducting a cross-sectional study on HCWs, we were able to determine that the majority of study participants experienced AEs after the Janssen COVID-19 vaccine. However, the AEs were largely perceived to be generally mild, neither requiring treatment nor staying for more than 24 h. Being young was significantly associated with having at least one AE. Our results support the safety profile of the Janssen vaccine, and could be used to build confidence in vaccine uptake in Tigray region.

In view of the success of the current vaccines against COVID-19 worldwide, it can therefore be concluded that the benefits outweigh the risks.

Most participants reported that they had experienced at least one AE after vaccination with the Janssen COVID-19 vaccine. The development of AEs as a result of vaccine reactogenicity due to the inflammatory response to vaccine components is a relatively common scenario^25^. In studies by Klugar M et al. and Agnes Kant et al. 88% and 82% of study participants reported AEs, respectively^26,27^. Similarly, as reported by Bijay Bhandari et al. in their report on AEs following administration of Janssen's COVID-19 vaccine, injection site pain and headache were the most common local and systemic AEs, observed in our study, respectively^9^. These findings support the results of the clinical trials conducted to evaluate the safety and efficacy of the Janssen COVID-19 vaccine^4^.

Most of the AEs experienced by the study participants were perceived as mild to moderate. Other studies also support our findings. A Jordanian study examining the side effects of vaccines in HCWs found that most reported side effects were of mild to moderate severity^28^. An online survey in Valencia, Spain, also found that the majority of side effects reported by study participants were mild to moderate in severity^29^. Janssen's COVID-19 vaccine has been associated with several serious AEs, including thrombosis with thrombocytopenia syndrome and anaphylactic reaction^8,30^. No hospitalizations, deaths, or other serious AEs occurred in our study, with the exception of two HCWs who reported anaphylactic reaction, four participants who developed a urticarial- like reaction, and one person who reported transient arm weakness. None of the AEs that occurred were reported to the immunization program or the Ethiopian Food and Drug Administration. This is a testament to the war related disintegration of health infrastructure and surveillance system in the region. Following a safety review, the FDA and the U.S. Centers for Disease Control and Prevention determined that Janssen's COVID-19 vaccine is safe and can be administered as an alternative to Pfizer and Moderna in certain populations^31^. The current study supports this position, although it is important to note that due to the small sample size in this study, it is difficult to draw conclusions about the actual prevalence of serious AEs associated with the Janssen COVID-19 vaccine. The efficacy of the vaccine in preventing moderate to severe COVID-19 infections, hospitalizations and deaths is acceptable^4^. Although adenovirus-based vaccines such as the Janssen vaccine have lower efficacy compared to mRNA-based vaccines, they are less expensive and less demanding to store^12^. Therefore they constitute a better option in resource-limited settings like our setup, and can play a pivotal role in curtailing the pandemic.

In line with other studies, a significant proportion of participants reported that their symptoms did not warrant treatment. An analysis of a large data pool from the United States Vaccine Adverse Effect Reporting System Data by Amninder Singh et al. found that most of the solicited AEs did not require treatment^30^. In the current study, most of the AEs occurred within one day of vaccination, and no participant reported onset of AEs later than three days after vaccination. Furthermore, AEs were transient, and disappeared within one day in the majority of the HCWs. These results are consistent with those of Lounis et al. and Chongliang et al. In these studies, most AEs occurred within 24 h of vaccination, and did not last longer than 48 h^32,33^. Our results suggest that the AEs reported after taking the Janssen COVID-19 vaccine were not alarming, and do not call into question the need for vaccination. In light of these findings, it is essential to encourage HCWs in particular and the population in general to get vaccinated, and follow the vaccination recommendations. This is necessary and should be emphasized especially in areas with communication deficits, where misconceptions about vaccines can be taken at face value because guidelines and expert recommendations have difficulty reaching the public.

This study showed that younger age was significantly associated with at least one AE. Several studies have shown that aging decreases the immune response^29^. Therefore, our finding is not surprising, as the AEs that occurred after vaccination in this study were largely due to immunologic reactogenicity. A study conducted among dentists in the United States corroborates our findings and reports that more AEs occurred in individuals younger than 55 years^10^. As the elderly are at a significant risk of morbidity and mortality due to COVID-19, it is encouraging that the current vaccines against COVID-19 are safer in the elderly population.

In our study, however, no significant correlation was found between gender and the occurrence of AEs. This is in contrast to other studies that show a significant association between female gender and the occurrence of AEs. An online survey in Spain found that female gender was associated with a significant risk for the occurrence of AEs after administration of the Janssen COVID-19 vaccine^34^. In addition, variables such as previous COVID-19 infection and previous vaccination with other COVID-19 vaccines were not found to be significantly associated with the occurrence of AEs, although other studies have found a significant association between these factors and the occurrence of AEs after COVID-19 vaccination^27^. The small sample size may have downplayed the role of sex and other variables as important factors in the development of AEs in our study. Although vaccination in Tigray is currently stalled due to the siege, we recommend that the Tigray Regional Health Bureau and other humanitarian organisations develop a mechanism for population-wide tracking of AEs and associated factors once vaccine rollout begins.

This study will play an important role in combating the myths and misconceptions circulating around COVID-19 vaccines and convincing the general population of the importance of vaccination in combating the COVID-19 pandemic under the conditions of communication blackout and collapse of the health information system in Tigray, Northern Ethiopia. The data in our study was collected in person, unlike most studies where AEs were recorded online. This creates a room for recording symptoms that may not be easily recognized by study participants, especially those with lower levels of education. However, our study also has its limitations. Given the time gap between the days of vaccination and data collection, and the fact that the study is based on self-reported AEs , participants may not have provided accurate information on AEs due to recall errors. In addition, the study did not include follow-up for the occurrence of medium- and long-term AEs of the vaccine. Furthermore, the generalizability of the study could be impaired as only medical personnel were involved, the participants were young, and the study was conducted in only one institution. In addition, cross-sectional studies cannot establish a cause–effect relationship between the administration of the vaccine and AEs, as it is a snapshot in nature, which may lead to recall bias and under- or over-reporting of AEs. The study population may not be representative of all HCWs and the general population in the region, leading to potential bias in the results. Cross-sectional studies may also not capture delayed or long-term AEs due to the short-term nature of the observations.

This study also assumes that the HCW population was homogenous in terms of demographics, health status, and vaccine acceptance, which may not always be the case. It also assumes that participants accurately report their AEs without intentional or unintentional misrepresentation and any AEs observed are directly attributable to the Janssen vaccine without considering other confounding variables.

## Conclusions

This study found that most of the study participants experienced AEs following administration of the Janssen COVID-19 vaccine. The most common local AE was pain at the injection site, while headache predominated among the systemic AEs. Younger age was significantly associated with the occurrence of AEs. The majority of study participants reported that most AEs were mild to moderate in severity. In addition, most AEs were transient and did not require medical attention.

Overall, our study has shown that the vaccine is associated largely with minor AEs. We therefore recommend that HCWs in particular and the population in general be vaccinated.

### Supplementary Information

Supplementary Information.

## Data Availability

All data relevant to the study are included in the manuscript or can be shared upon request. The corresponding author can be contacted in case someone wants to request the data from this study.
