# Supplementary material for: Assessment of adverse events among healthcare workers following the Janssen COVID-19 vaccine in Tigray, Ethiopia
Source: Sci Rep. 2024 Feb 8;14:3255. doi: 10.1038/s41598-024-53561-8 (PMC10853211; doi:10.1038/s41598-024-53561-8)
Supplement: Supplementary file 1 — Supplementary Information. [file 41598_2024_53561_MOESM1_ESM.docx]

**Informed consent form**

I have read (it was read to me) the participant information sheet. I have clearly understood the purpose of the research, the procedure, the risks and benefits, issues of confidentiality, the rights of participating, and the contact for any queries. I have been given the opportunity to ask questions for things that may have been unclear. I have informed that the right to withdraw from the study at any time or not to answer any question that I do not want. Therefore, I declare my voluntary consent to participate in this study with my initials (signature) as indicated below.

Participant signature_______________ Date_______________

Name of data collector______________________ Signature__________

**Contact address:** If you have any question you can contact at any time with phone number**:** **+**251949711684 (address of principal investigator). Email: **Email:** Bis9live@gmail.com

**1. Sociodemographic characteristics**

| No. | Question | Response | Remark |
| --- | --- | --- | --- |
| 1 | Code |  |  |
| 2 | Gender | 1. Female 2. Male |  |
| 3 | Religion | 1. Christian 2. Muslim 3. Other, mention_______ |  |
| 4 | Marital status | 1. Single  2. Married  3. Divorced  4. Widowed  5. Other, specify_______________ |  |
| 4 | Occupation | 1. Medical doctor 2. Doctor of dental surgery/medicine (DDS/DDM) 3. Nurse/midwifery 4. Pharmacist 5. Pharmacy technologist 6. Radiology technologist 7. Radiology technician 8. Laboratory technologist 9. Laboratory technician 10. Anesthetist 11. Physiotherapist 12. Supportive staff (Cleaner, porter, oxygen attendant, social worker, guard, ambulance driver, data encoders at triage, wards and ICU) 13. Other, mention___ |  |
| 5 | Educational background | 1. Doctorate degree 2. Masters 3. Degree 4. Diploma 5. Secondary school 6. Primary school 7. Elementary school 8. Read and write 9. Illiterate |  |
| 8 | Pregnancy status | 1. Pregnant 2. Not pregnant 3. NA |  |
| 9 | BMI (body mass index) |  |  |

**2. Comorbidities**

| No. | Question | Response | Remark |
| --- | --- | --- | --- |
|  | HIV | 1. yes  2. no |  |
|  | Diabetes | 1. yes  2. no |  |
|  | Tuberculosis | 1. Yes  2. No |  |
|  | CKD | 1. yes  2. no |  |
|  | CLD | 1. yes  2. no |  |
|  | Hypertension | 1. yes  2. no |  |
|  | Other cardiovascular disorder | 1. yes  2. no |  |
|  | History of Stroke before vaccination | 1. yes  2. no |  |
|  | History of thromboembolism before vaccination | 1. yes  2. no |  |
|  | History of allergy before vaccination | 1. yes  2. no |  |
|  | B. asthma | 1. yes  2. no |  |
|  | Do you smoke? | 1. Yes  2. N1.o |  |
|  | Do you have medications you currently take? | 1. Yes  2. No |  |
|  | If yes to Qn 13, please mention |  |  |
|  | Have you been taking high-dose steroids ? | 1. Yes  2. No |  |
|  | Have you been taking chemotherapy? | 1. Yes  2. No |  |
|  | Have you been taking other immunosuppressive agents? | 1. Yes  2. No |  |
|  | If yes to Qn 17, please mention. |  |  |

**3. Previous vaccination history and COVID-19 status**

| No. | Question | Response | Remark |
| --- | --- | --- | --- |
|  | Previous laboratory confirmed COVID-19? | 1. Yes  2. No |  |
|  | Laboratory confirmed COVID-19 just before the current vaccination? | 1. Yes  2. No |  |
|  | Presence of COVID-19 like symptoms just before the current vaccination? | 1. Yes  2. No |  |
|  | Did you receive the AstraZeneca vaccine previously? | 1. Yes  2. No |  |
|  | If yes, how many doses? | 1. one  2. two |  |
|  | If yes to Qn 4, did you witness any side effects following AstraZeneca? | 1. yes  2. no |  |
|  | If yes, how do you put the severity of the side effects when compared to the current J&J vaccine? | 1. Less severe  2. Similar  3. More severe  4. Not appropriate question for me | You choose 4 If no side effects of J &J vaccine |
|  | Did you take other vaccines before taking J & J vaccine? | 1. Yes  2. No |  |
|  | If yes to Qn 8, please mention the type of vaccine you took? |  |  |

**4. Did you witness side effects following J & J vaccine?**

1. Yes

2. No

If yes, answer the following questions.

**5. Local side effects**

| No. | Question | Response | Remark |
| --- | --- | --- | --- |
|  | Injection site pain | 1. yes  2. no |  |
|  | Injection site erythema | 1. yes  2. no |  |
|  | Injection site pruritus | 1. yes  2. no |  |
|  | Injection site swelling | 1. Yes  2. No |  |
|  | Rash at the injection site | 1. Yes  2. No |  |
|  | Injection site discharge | 1. yes  2. no |  |
|  | Local lymph node enlargement after vaccination | 1. Yes  2. No |  |

**6. Systemic side effects**

| No. | Symptoms | Response | Remark |
| --- | --- | --- | --- |
|  | Headache | 1. yes 2. no |  |
|  | Chills | 1. yes  2. no |  |
|  | Fever | 1. yes  2. no |  |
|  | Fatigue | 1. yes  2. no |  |
|  | Arthralgia | 1. yes  2. no |  |
|  | Myalgia | 1. yes  2. no |  |
|  | Dizziness | 1. yes  2. no |  |
|  | Nausea | 1. yes  2. no |  |
|  | Vomiting | 1. yes  2. no |  |
|  | Diarrhea | 1. yes  2. no |  |
|  | Bilateral fascial weakness | 1. yes  2. no |  |
|  | Shortness of breath | 1. yes  2. no |  |
|  | Chest pain | 1. yes  2. no |  |
|  | Weakness of one side of the body | 1. yes  2. no |  |
|  | Bilateral lower extremity weakness | 1. yes  2. no |  |
|  | Weakness of one extremity | 1. yes  2. no |  |
|  | Eye swelling/edema | 1. yes  2. no |  |
|  | Face swelling/edema | 1. yes  2. no |  |
|  | Pharyngeal swelling/edema | 1. yes  2. no |  |
|  | Lip swelling/edema |  |  |
|  | Abdominal swelling/extremity swelling | 1. yes  2. no |  |
|  | Foamy urine | 1. yes  2. no |  |
|  | Decreased urine output | 1. yes  2. no |  |
|  | Hypertension | 1. yes  2. no |  |
|  | Hypotension | 1. yes  2. no |  |
|  | Diagnosis of bell’s palsy | 1. yes  2. no |  |
|  | Diagnosis of anaphylactic reaction | 1. yes  2. no |  |
|  | Diagnosis of myocarditis/pericarditis | 1. yes  2. no |  |
|  | Diagnosis of Guillain-Barre syndrome | 1. yes  2. no |  |
|  | Diagnosis of Transverse myelitis | 1. yes  2. no |  |
|  | Diagnosis of Thrombosis with thrombocytopenia syndrome (TTS) | 1. yes  2. no |  |
|  | Diagnosis of Nephrotic syndrome | 1. yes  2. no |  |
|  | Diagnosis of Syncope | 1. yes  2. no |  |
|  | How many hours after the vaccination did the symptom/s appear? |  |  |
|  | For how long did the symptom/s stay (hours)? |  |  |

**7. Severity of symptoms**

| No. | Question | Response |  |
| --- | --- | --- | --- |
| 1 | How do you put the severity of symptoms? | 1. Very mild  2. Mild  3. Moderate  4. Severe  5. Very severe |  |
| 2 | Did the side effects require treatment? | 1. Yes  2. No |  |
| 3 | If Yes, what type of treatment? | 1. Home based treatment  2. Took over the counter medication  3. Consultation with a health professional  4. Visit to the emergency  5. Admission to ward  5. Admission to ICU |  |
